# Input precision, output excellence: the importance of data quality control and method selection in disease risk mapping —authors’ reply

**DOI:** 10.1016/j.lanepe.2024.100947

**Published:** 2024-05-24

**Authors:** Zia Farooq, Joacim Rocklöv, Jonas Wallin, Najmeh Abiri, Maquines Odhiambo Sewe, Henrik Sjödin, Jan C. Semenza

**Affiliations:** a Department of Public Health and Clinical Medicine, Section of Sustainable Health, Umeå University, Sweden; b Heidelberg Institute of Global Health and Interdisciplinary Center for Scientific Computing, University of Heidelberg, Im Neuenheimer Feld 205, Heidelberg 69120, Germany; c Department of Statistics, Lund University, Sweden

We would like to thank Taheri et al., for critically examining the datasets that underpin our analysis of eco-climatic drivers of West Nile Virus (WNV) transmission in Europe.^1^^,^^2^ They aptly note that besides eco-climatic variables, our entomological and ornithological data failed to significantly enhance our WNV transmission model. While this seems to contradict the established association between avian and vector abundance and WNV incidence, as noted by Taheri et al., we report that eco-climatic variables had high discriminatory power (AUC > 93%, accuracy > 90%) to classify a WNV outbreak area.^2^ At first glance, these findings seem to contradict our mechanistic understanding of WNV transmission, but as we elaborate below, there is a rationale why our model works well.

Taheri et al., astutely observe the absence of vector data for numerous NUTS3 regions, despite the suspected widespread presence of *Culex pipiens* and *Culex modestus* across Europe. It is important to note that we limited our main analysis to regions with more complete information on vector abundance data, censuring those NUTS3 where data were missing. While our model acknowledges vector abundance as a significant predictor, their importance ranks below eco-climatic drivers. Thus, on a European scale, the relatively weak association between vector abundance and regions with WNV infections in humans did not appear to be explained by a lack of vector data alone.

Taheri et al., also correctly noticed that *Cx. modestus* was missing in our database in 2018, but still scored as a predictor in our model for that year. This seeming inconsistency is due to the fact that 2018 data were withheld from model fitting; we mapped features with annual scale from the preceding year (2017) to predict WNV transmission in 2018 (see subsection ‘Model selection’ of Methods section).^2^ Taheri et al., highlight the risk for confirmatory bias but machine-learning algorithm test a wide range of drivers and predictors not singling out one or a few for statistical testing. Frameworks like explainable AI are developed to explain what features are predictive. As for the avian hosts, European-wide databases are not available that accurately reflect the distribution of birds susceptible to or infected with WNV, and their seasonal patterns.

We tested over 100 features and over 11,000 interactions to produce a model that is highly predictive, with out-of-sample (external) data withheld from model fitting, based on several validation techniques.^2^ Our findings indicate that high resolution vector and host (avian) data may not be necessary for a highly predictive WNV model if eco-climatic features can compensate for vector and avian data and their interactions. Our model indicates that these eco-climatic features and the limited vector and host data, are sufficient for the spatio-temporal prediction of WNV outbreaks in Europe at the time scale and level of accuracy presented in the paper.^2^

The rationale why our eco-climatic WNV model, even with limited vector and avian data, is so predictive might be based on the following. The eco-climatic features themselves determine, at least in part, vector and avian abundance and it is likely that eco-climatic drivers capture part of this variability, especially variability between years. The year-to-year variability in outbreaks may ultimately be driven by more distal features mediated by vector and avian data. In fact, we showed that even with the lack of high-quality vector and avian data, it is possible to make accurate WNV predictions at this scale, which can be operationalized by public health authorities. By circumventing the bottleneck of high-resolution vector and host data, resource-strapped regions can deploy our model for outbreak prevention and response. Our findings question the premise brought forward by Taheri et al., that precision input data are indispensable for a highly predictive WNV risk model.^1^

We hope to have convinced Taheri et al., that we have adequately addressed biases and errors in our input variables to avoid the principle of “garbage-in, garbage-out”. Such a connotation does not do our model justice, particularly in light of its high predictive power of a massive outbreak year, such as 2018. We eagerly anticipate their model with complete and high resolution avian and vector abundance data that outperforms the predictive power of ours at the European scale. Such a comparative assessment of their European-wide WNV model against ours, fosters a constructive dialogue and advances our understanding of WNV transmission dynamics in Europe.

## Contributors

JCS wrote the first draft; JR, ZF, JW and HS edited the draft; all authors approved the final version.

## Declaration of interests

None.
